# Heterogeneous Reactions of α-Pinene on Mineral Surfaces: Formation of Organonitrates and α-Pinene Oxidation Products

**DOI:** 10.1021/acs.jpca.2c02663

**Published:** 2022-06-16

**Authors:** Eshani Hettiarachchi, Vicki H. Grassian

**Affiliations:** Department of Chemistry and Biochemistry, University of California San Diego, 9500 Gilman Drive, La Jolla, California 92093, United States

## Abstract

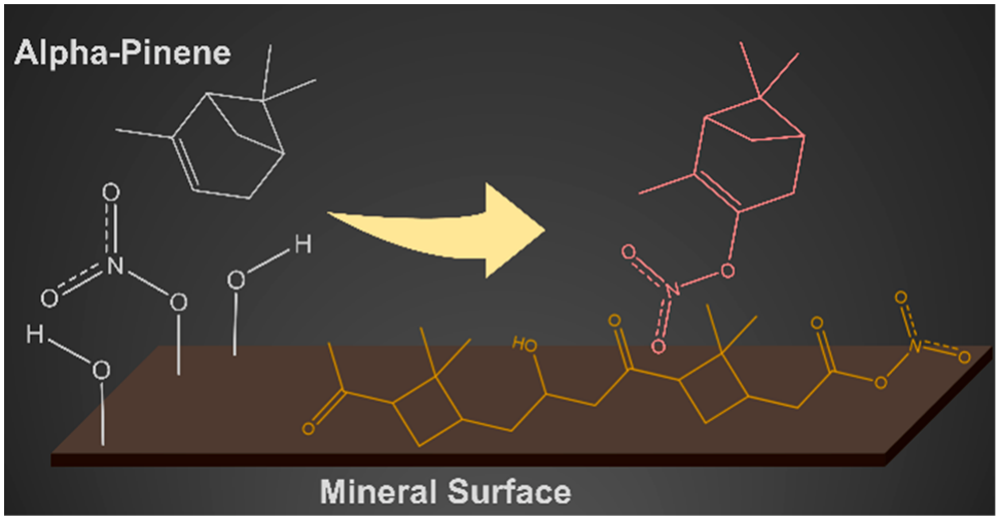

Organonitrates (ON) are important components of secondary organic aerosols (SOAs). α-Pinene (C_10_H_16_), the most abundant monoterpene in the troposphere, is a precursor for the formation of several of these compounds. ON from α-pinene can be produced in the gas phase via photochemical processes and/or following reactions with oxidizers including hydroxyl radical and ozone. Gas-phase nitrogen oxides (NO_2_, NO_3_) are N sources for ON formation. Although gas-phase reactions of α-pinene that yield ON are fairly well understood, little is known about their formation through heterogeneous and multiphase pathways. In the current study, surface reactions of α-pinene with nitrogen oxides on hematite (α-Fe_2_O_3_) and kaolinite (SiO_2_Al_2_O_3_(OH)_4_) surfaces, common components of mineral dust, have been investigated. α-Pinene oxidizes upon adsorption on kaolinite, forming pinonaldehyde, which then dimerizes on the surface. Furthermore, α-pinene is shown to react with adsorbed nitrate species on these mineral surfaces producing multiple ON and other oxidation products. Additionally, gas-phase oxidation products of α-pinene on mineral surfaces are shown to more strongly adsorb on the surface compared to α-pinene. Overall, this study reveals the complexity of reactions of prevalent organic compounds such as α-pinene with adsorbed nitrate and nitrogen dioxide, revealing new heterogeneous reaction pathways for SOA formation that is mineralogy specific.

## 1 Introduction

Aerosols are known to play a key role in climate and air quality, with high concentrations leading to adverse health effects (e.g., cardiovascular disease, lung cancers).^1−4^ Aerosols also cause reduced visibility and haze conditions associated with urban air pollution.^5^ α-Pinene, C_10_H_16_, is the most abundant atmospheric monoterpene with an average estimated emission of 66 Tg yr^–1^. It is an important precursor to several compounds that lead to the formation of secondary organic aerosols (SOAs).^6−9^ These compounds include oxygenated organic compounds found in polluted atmospheric conditions as well as organosulfates, ROSO_3_H (OS), and organonitrates, RONO_2_ (ON), all contributing to SOA formation.^10−14^ These α-pinene-derived compounds have been detected in ambient air.^15−18^

Atmospheric ON formation occurs by either OH radical-initiated photochemical reactions or NO_3_ radical-initiated nocturnal reactions of anthropogenic and biogenic volatile organic compounds. These reactions can produce ON compounds with vapor pressures low enough to condense and form SOA.^19−23^ In one such study, the formation of a series of ON compounds from reactions of α-pinene with NO_3_ radicals was observed.^22^ In another study, the photochemistry of the hydroxyl radical oxidation of α-pinene under high NO_*x*_ conditions was studied and the formation of a range of ON compounds from α-pinene in the presence of nitric oxide (NO) was reported.^24^ In addition, reactions of α-pinene with atmospheric oxidizers, such as O_3_ and OH radicals lead to the formation of a range of α-pinene oxidation products that are known as first generation oxidation products.^4,6,25,26^ Several of these products have been identified as pinonaldehyde, pinonic acid, and α-pinene oxide, as well as a structurally diverse set of organic peroxides. These oxidation products can also act as a reactant in generating atmospheric ON compounds.^27^ These oxidation reactions are generally formed in the daytime, suggesting the formation of ON compounds from α-pinene primarily occurs in the presence of atmospheric oxidizers and/or photochemistry.^19,28^ All of these studies are primarily focused on understanding SOA formation from gas-phase condensation.

In this study, heterogeneous reactions of α-pinene nitrogen oxides on iron oxide (α-Fe_2_O_3_) and kaolinite (SiO_2_Al_2_O_3_(OH)_4_) surfaces are investigated to understand heterogeneous SOA formation on mineral dust aerosol. Both iron oxides and kaolinite are reactive components of mineral dust aerosol.^29^ Heterogeneous chemistry of gas-phase species, both organic and inorganic components with atmospheric mineral dust aerosols, have been widely studied. For instance, adsorption of α-pinene on different types of silica surfaces in the absence of photochemistry showed reversible adsorption.^30,31^ Additionally, the adsorption of NO_2_ and HNO_3_ on mineral dust has been extensively investigated.^32−35^ NO_2_ is emitted to the atmosphere primarily via fossil fuel combustion and vehicle exhausts.^36−39^ Because of the higher correlation of atmospheric NO_2_ concentrations to traffic, it is used as a traffic-related air pollution marker.^38^ Furthermore, NO_2_ concentrations vary significantly over shorter distances in shorter timeframes.^38,39^ Previous studies have shown that mineral surfaces exposed to NO_2_ yield two major surface-adsorbed species (nitrate and nitrite) thereby producing a surface for organic compounds such as α-pinene to interact with and facilitate the formation of ON. Similarly, gas-phase HNO_3_ acid reacts on mineral dust particles to yield adsorbed nitrates and in some cases molecular nitric acid. Despite this knowledge, little is known about the ability of mineral dust surfaces to mediate and catalyze ON formation. Therefore, the role of surface chemistry in reactions of α-pinene with atmospheric NO_2_ and HNO_3_ on iron oxide (hematite, α-Fe_2_O_3_) and kaolinite (SiO_2_Al_2_O_3_(OH)_4_) surfaces is investigated here. The experiments in the present study were conducted under dark conditions to better understand the underlying role of the mineral surface. For these studies, both Fourier transform infrared (FTIR) spectroscopy and high-resolution mass spectrometry (HRMS) were used to better understand the chemistry of α-pinene on these atmospherically relevant surfaces.

## 2 Materials and Methods

### 2A Transmission FTIR Experiments and Methods

Transmission FTIR spectroscopy was used to study the adsorption of α-pinene on hematite at 296 ± 1 K. Additional details of this system have been previously described.^40−44^ The mineral particles (hematite, α-Fe_2_O_3_, 99+%, Fisher Scientific or kaolinite, SiO_2_Al_2_O_3_(OH)_4_, Sigma-Aldrich) with a BET surface area of 80 ± 10 and 8.4 ± 0.5 m^2^/g, respectively, were heated in an oven at 200 °C overnight to remove organic contaminants and then pressed onto one-half of a tungsten grid (ca. 5 mg). The grid was then placed in the sample IR cell compartment, held by two stainless steel jaws. Following the preparation of the mineral sample and placement in the IR cell, the system was evacuated for 4 h using a turbomolecular pump. The mineral sample was subsequently exposed to 50% RH water vapor for 2 h to hydroxylate the surface. Once hydroxylated, the system was evacuated for another 6 h to remove water vapor in the chamber. After evacuation, the sample was exposed to the desired pressure of α-pinene (99+%, Sigma-Aldrich) for 20 min under dry conditions (RH < 1%). The α-pinene sample was degassed at least three times with consecutive freeze–pump–thaw cycles prior to use.

Reactions of NO_2_ (26.5 ppm in N_2_, Airgas) with α-pinene on mineral surfaces were studied. First, α-pinene was allowed to adsorb on the mineral surface for >20 min. The desired pressure of NO_2_ (7 mTorr) was then introduced into the IR cell. FTIR spectra were collected over a 4 h period through both halves of the tungsten grid to monitor gas-phase and surface-adsorbed α-pinene. Following adsorption, the system was evacuated overnight. The reactions of surface-adsorbed nitrates with α-pinene were then studied. First, to obtain a nitrated mineral surface, the mineral surface was introduced to nitric acid vapor taken from a concentrated mixture of H_2_SO_4_ (∼96 wt %)/HNO_3_(∼70 wt %) in a 3:1 ratio.^45^ The mineral surface was exposed to nitric acid vapor for 4 h and evacuated overnight. Following nitration, α-pinene was introduced to these modified mineral surfaces and the reactions were monitored for over 4 h. The experiments with HNO_3_ were conducted in a different experimental but with a very similar setup (instead of stainless steel a Teflon coated cell and a glass system was used).

Prior to and following the exposure to α-pinene, single-beam spectra (250 scans) of the surface and gas phase were acquired using a resolution of 4 cm^–1^ and covering the spectral range 600–4000 cm^–1^. For kinetic studies, spectra were acquired every 15 s for the first 15 min of the adsorption. Absorption spectra of α-pinene on mineral particles are reported as the difference in the mineral spectra before and after exposure to α-pinene. Absorption bands because of gas-phase α-pinene, measured through the blank half of the tungsten grid, were subtracted to obtain FTIR spectra of adsorbed α-pinene only.

### 2B HRMS Experiments and Methods

Organic products formed on mineral surfaces following reactions of α-pinene with NO_2_ and adsorbed nitrates in the dark were analyzed using a direct-injection linear ion trap (ThermoFisher Orbitrap) high-resolution mass spectrometer (HRMS). Adsorbed products were extracted from the hematite or kaolinite solid substrate using methanol (CH_3_OH, Fisher Scientific, HPLC grade) as the solvent. The sample vial, syringe, and all other glassware used in the transfer process were cleaned prior to use with methanol, and Milli-Q water (Millipore Sigma, 18.2 MΩ), and baked in an oven at 500 °C to further remove trace organics. Plastic vials used in sample preparation were sonicated in methanol for 60 min, and washed thoroughly prior to use. All of the samples were stored at −20 °C and analyzed within 24 h of collection. Separate experiments with approximately 100 mg of hematite were conducted for α-pinene adsorption on hematite, and NO_2_ + α-pinene reaction on hematite for extraction and HRMS detection.

HRMS analysis in both positive electrospray ionization (ESI) and negative ESI modes was used, although the detected ions for the reactions were observed in positive ESI mode ([M + H]^+^). The heated electrospray ionization (HESI) source was operated at 100 °C. The ESI capillary was set to a voltage of 3.5 kV at 350 °C. The HESI-Orbitrap MS was calibrated prior to use. Mass spectra were acquired with a mass range of 50–2000 Da. Peaks with mass tolerance of >2 ppm were rejected. Compositions were calculated with the following element ranges: 12C, 0–60; 1H, 0–100; 16O, 0–10; 14N, 0–5; 23Na, 0–5; 39K, 0–5; 56Fe, 0–5. Tandem mass spectrometry (MS/MS) with collision energy of 40 eV was used for structure determination.

## 3 Results and Discussion

### 3A α-Pinene Adsorption on Hematite and Kaolinite Surfaces at 296 K

**Figure 1:**
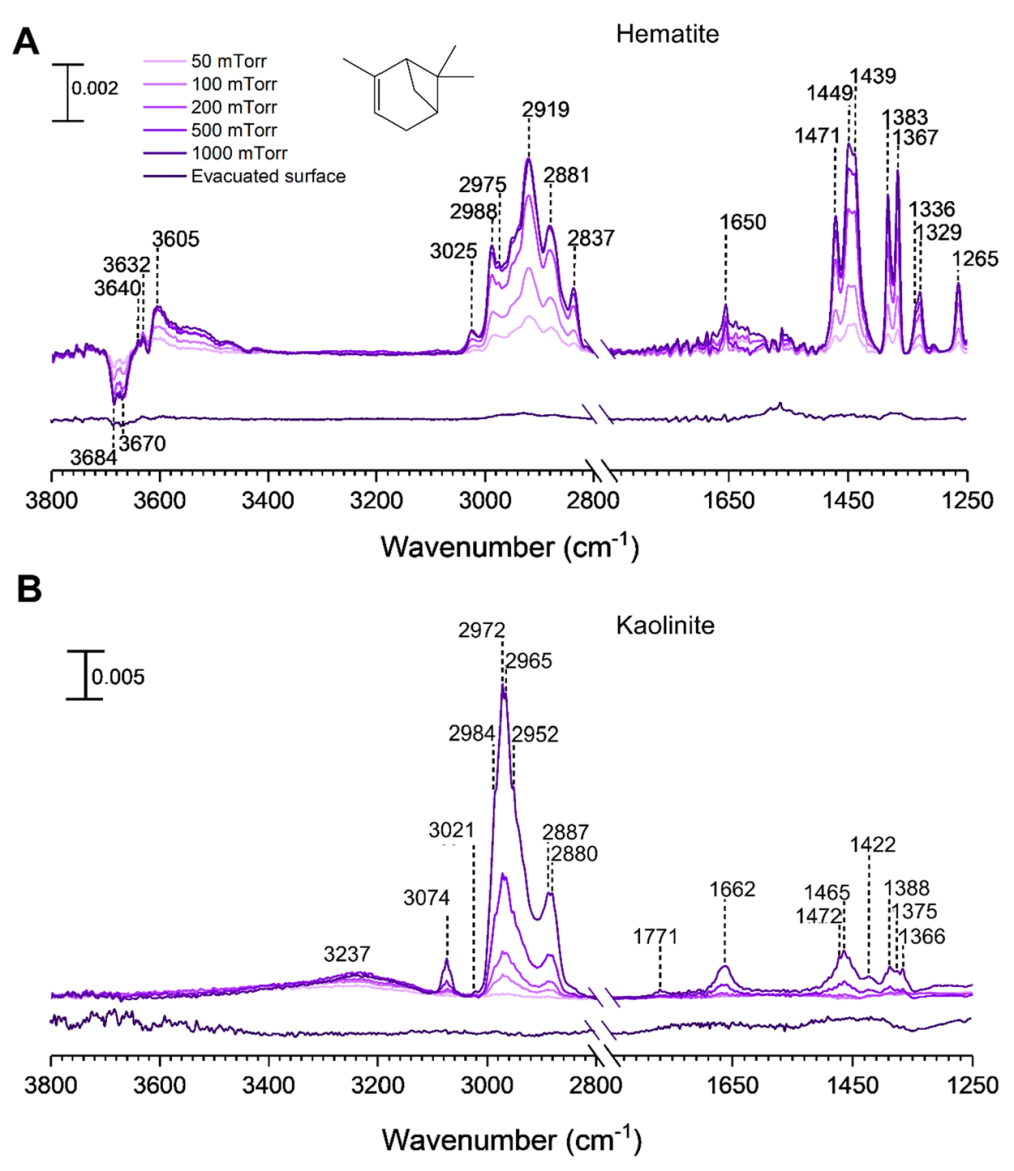
FTIR spectra of α-pinene adsorbed on (A) hematite and (B) kaolinite as a function of α-pinene pressure in the spectral regions from 2800 to 3800 cm^–1^ and from 1250 to 1500 cm^–1^. The absorbance scale is shown in the upper left for each surface.

Mineral particle surfaces exposed to gas-phase α-pinene at various pressures under dry conditions show new spectral features because of surface adsorption, as seen in Figure 1. Most notable are the infrared absorption bands in the regions extending from 2800 to 3800 cm^–1^ and from 1250 to 1500 cm^–1^. The bands between 2800 and 3100 cm^–1^ were assigned to various C—H stretching vibrations and the bands between 1300 and 1500 cm^–1^ were assigned to various C—H bending modes. (Table 1).^30,46,47^

**Table 1:** FTIR Peak Assignments of Adsorbed α-Pinene on Hematite and Kaolinite^30,46,47,49^

Peak Frequencies		
Hematite	Kaolinite^a^	Peak Assignments
		
1329, 1336, 1367, 1383, 1439, 1449	1329, 1366, 1449	aliphatic CH_2_ and CH_3_ bending, twisting and wagging modes
1471	1472	vinyl C—H bending mode
1650	n.o.^b^	C=C stretching mode
		
		
2837, 2881, 2919, 2975, 2988	2880, 2887, 2952, 2965, 2972, 2984	aliphatic CH_2_, and CH_3_ stretching modes, and Fermi resonances
3025	3021	vinyl C—H stretching mode
		
		
3605, 3632, 3640	n.o.	surface O—H hydrogen bonded stretching mode
3670, 3684	n.o.	isolated surface O—H stretching mode

a Peak frequencies from the initial FTIR spectrum shown in Figure 2A.

b n.o. = not observed.

For hematite, the frequencies observed for the absorption bands found for adsorbed α-pinene corresponded closely to its gas-phase vibrational frequencies suggesting that α-pinene is molecularly adsorbed onto the hematite surface (Figure 1A).^30,48^ With increasing α-pinene pressure, there is an increase in intensity of bands at 3605, 3632, and 3640 cm^–1^ with a concomitant loss of surface hydroxyl groups on hematite at 3670 and 3684 cm^–1^. These can be attributed to the formation of π hydrogen bonds between α-pinene and the surface hydroxyl groups.^30^ The spectral feature around 1650 cm^–1^ was assigned to the C=C bond stretching mode of adsorbed α-pinene.^47^ Upon evacuation overnight, almost all of the adsorbed α-pinene is removed, thus suggesting reversible adsorption of α-pinene on hematite surfaces. Furthermore, HRMS analysis of samples extracted from α-pinene-treated surface detected only a minor quantity of residual α-pinene (C_10_H_17_*m*/*z* 137.13) in the positive ESI mode. No α-pinene derivatives were confirmed during the HRMS analysis. This indicates that adsorbed α-pinene does not transform into other compounds on the hematite surface under dark and dry conditions.

In contrast to the hematite surface, adsorption of α-pinene on kaolinite shows significant changes in the vibrational frequencies and intensities of absorption bands in the spectrum compared to the gas phase (Figure 1B). Most notable is the disappearance of the peak at 3021 cm^–1^ corresponding to the olefinic C—H stretch, indicating reactions of α-pinene (Figures 1A and 2B) with new peaks appearing at 3074, unsaturated C—H stretch and 1771 and 1662 cm^–1^ suggesting surface oxidation and the formation of C=C and C=O bonds.^30,46,49^

**Figure 2:**
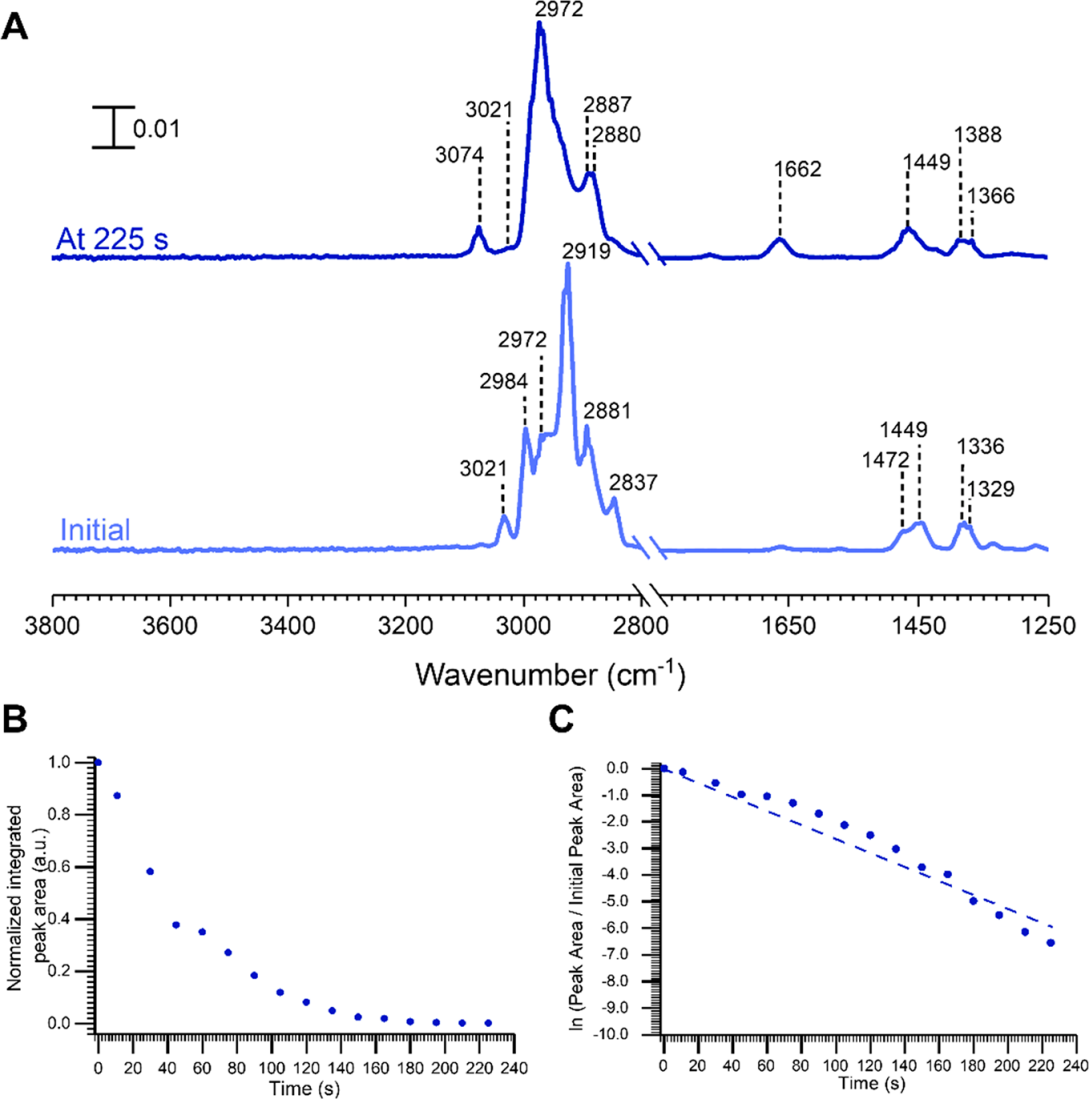
Kinetics of the surface reaction of α-pinene on kaolinite. (A) FTIR spectra at time *t* = 0 and at 225 s after adsorption. (B) Changes in the normalized peak area for the olefinic C—H stretch at 3021 cm^–1^ as a function of time. (C) First-order kinetic fit (*R*^2^ = 0.98) yields a first-order rate constant of 0.021 s^–1^.

#### Kinetics of α-Pinene Oxidation on Mineral Surfaces

To better understand the kinetics of reaction of α-pinene on kaolinite, the FTIR spectra collected within the initial 225 s were analyzed. The initial spectrum of α-pinene adsorbed on kaolinite contains peaks corresponding to the gas-phase α-pinene, indicating initial molecular adsorption (Figure 2A). At *t* = 225 s, new peaks at 3074 and 1662 cm^–1^ appear whereas the peak at 3021 cm^–1^ corresponding to the olefinic C—H stretch disappears. Furthermore, the peak area under the curve for the peak at 3021 cm^–1^ (for kaolinite) was calculated and normalized. The peak area was plotted against time. (Figure 2B). The kinetics of reaction of α-pinene on kaolinite for the time, *t*, between 30 and 120 s was calculated according to a pseudo first-order rate equation (eq 1) are shown in Figure 2C.
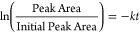
1

The rate of loss of adsorbed α-pinene, as shown by the disappearance of the peak at 3021 cm^–1^, was monitored and gave a rate constant, *k*, of 0.021 s^–1^.

#### Formation of Surface-Bound Products from the Adsorption of α-Pinene on Kaolinite

**Figure 3:**
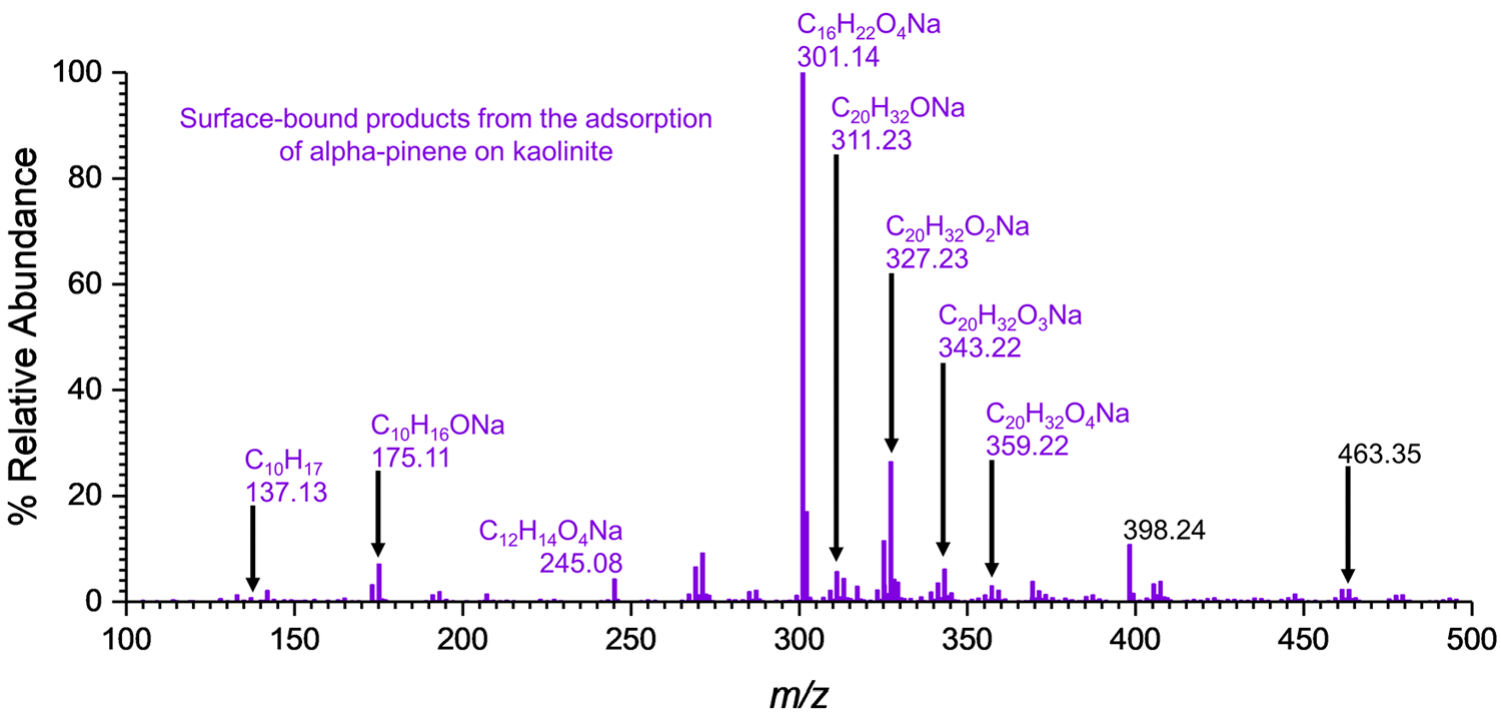
HRMS pattern of the surface-bound products formed on kaolinite upon exposure to α-pinene. The products were identified in positive ESI mode (M + H)^+^.

HRMS analysis of surface-bound products extracted from the kaolinite surfaces exposed to α-pinene shows two major products (Figure 3). These compounds were identified as **1**, C_20_H_32_O_4_Na (compound **1**), corresponding to a pinonaldehyde dimer (*m*/*z* = 359.22) and C_10_H_16_O_2_Na (compound **2**) corresponding to pinonaldehyde (*m*/*z* = 191.10). Therefore, it is proposed that α-pinene oxidizes to pinonaldehyde on the kaolinite surface and readily undergoes dimerization. Dimerization of pinonaldehyde yields two isomers, one via aldol condensation and the other by gem diol formation followed by subsequent dehydration.^50^ Because of the subsequent oxygen loss observed in the HRMS pattern with C_20_H_32_O_4_Na, C_20_H_32_O_3_Na, C_20_H_32_O_2_Na, and C_20_H_32_ONa, the aldol condensation product was assigned the structure shown for compound **1** (Table 2).

**Table 2:**
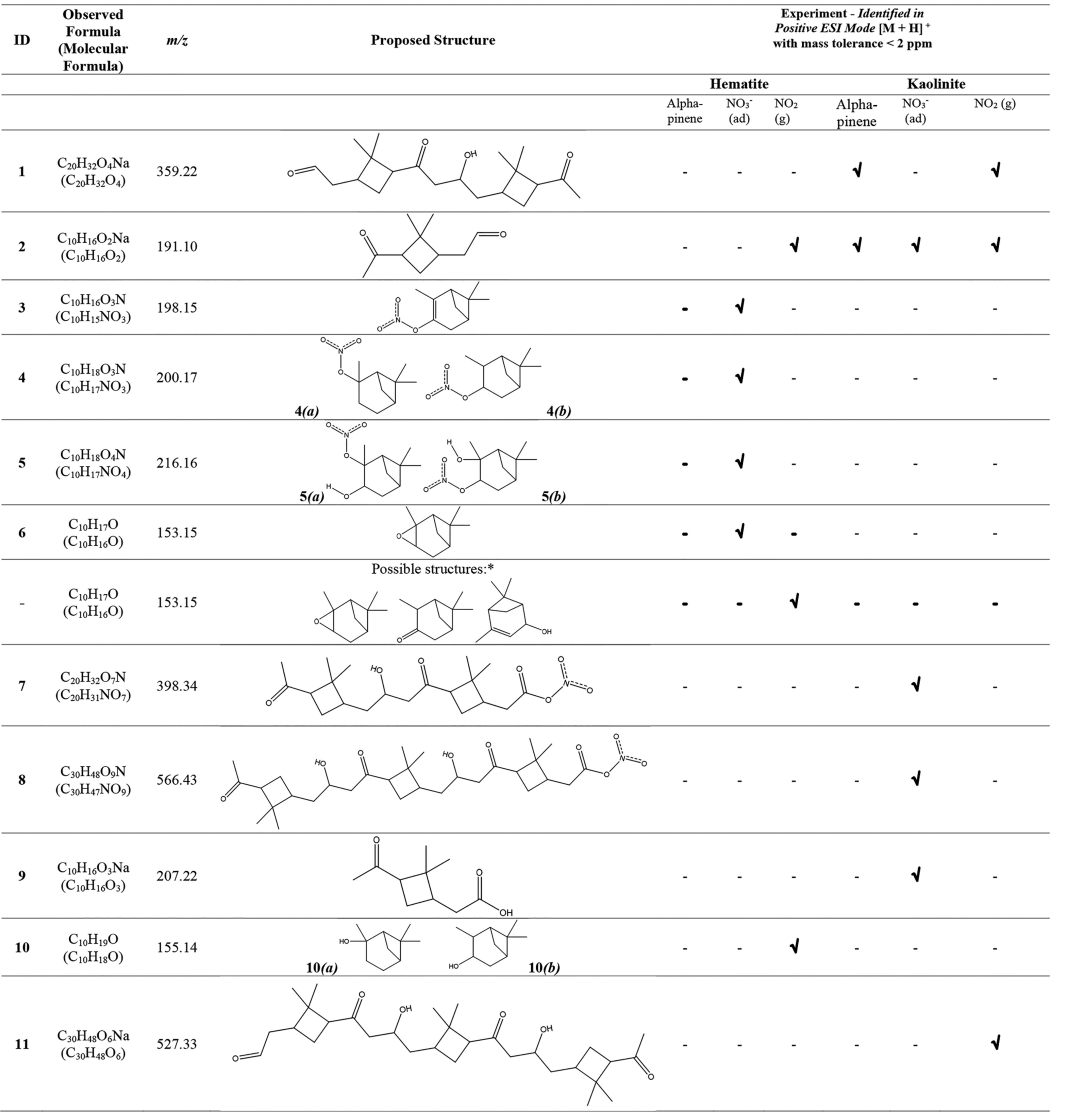
Identified Surface-Bound Products from HRMS Analysis Coupled with MS/MS^a^,^b^

a The * identifies proposed possible structures.

b All products were identified in positive ESI mode [M + H]^+^ with mass tolerance <2 ppm.

**Scheme 1:**
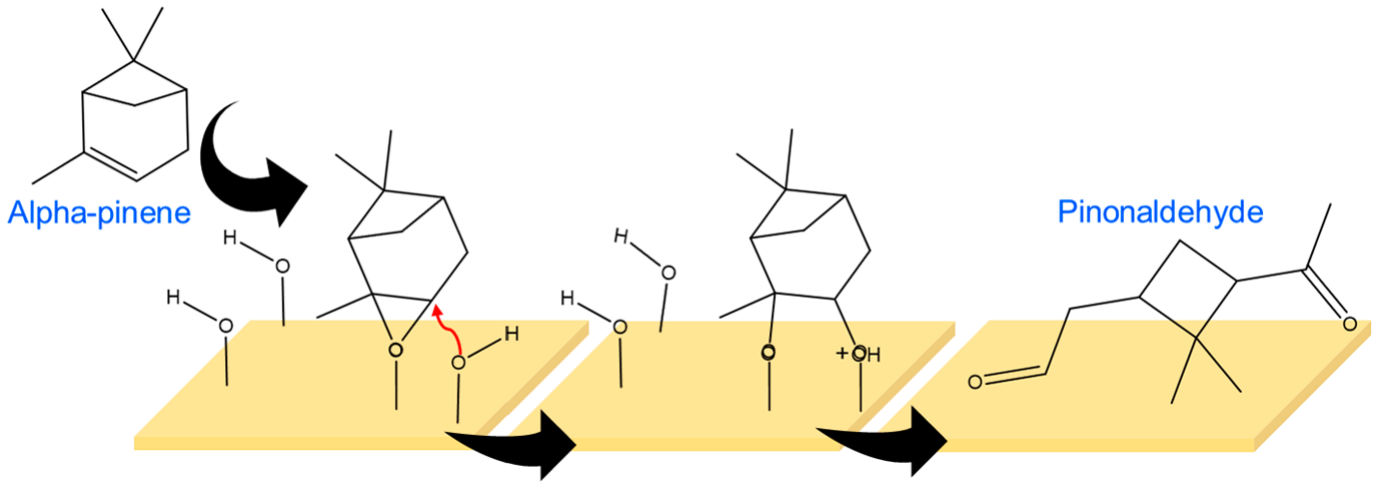
Proposed Mechanism for Oxidation of α-Pinene to Pinonaldehyde upon Exposure to Kaolinite Surface This proposed mechanism was adapted in part from Lederer et al. (2016).^51^

The formation of pinonaldehyde from α-pinene on kaolinite surfaces likely involves surface redox sites and surface hydroxyl groups. A possible mechanism involves dihydroxylation of the C=C bond from the surface redox sites and surface hydroxyl groups followed by a glycol cleavage. A similar dihydroxylation was observed previously for limonene, an isomer of α-pinene, on kaolinite.^51^ Furthermore, in our studies, a small HRMS peak at 193.12 corresponding to dihydroxylated α-pinene with the formula C_10_H_18_O_2_Na was observed supporting a possible surface hydroxyl groups driven oxidation pathway (Scheme 1). Furthermore, as calculated using Avogadro molecular building platform, α-pinene molecules have an average length perpendicular to C=C of 5.974 Å. The average basal distance of kaolinite between two layers is 7.2 Å,^52^ indicating that α-pinene molecules are small enough to interact with these interlayers, thereby possibly increasing the reactivity whereas the crystalline surface of hematite may facilitate only the physisorption of α-pinene. Therefore, these findings underscore the importance of the mineral surface specificity in forming oxidation products and warrant further investigation.

### 3B Reactions of Adsorbed Nitrates and Gas-Phase NO_2_ with α-Pinene

In FTIR experiments, the mineral surfaces were first nitrated by exposure to nitric acid. For nitrated hematite and kaolinite surfaces, several coordination modes of adsorbed HNO_3_ and NO_3_^–^ were observed, as has been previously discussed in detail (see Figure S1 in the Supporting Information).^33,44,53−56^ It can be seen that the nitrate coordination differs on these surfaces. Briefly, multiple coordination modes (monodentate, bidentate, and bridging) were observed on nitrated hematite surface.^44,57^ In contrast, however, adsorbed nitrate on kaolinite had spectral features more closely associated with water solvated nitrate.^44,57^ Several new surface products formed following the adsorption of α-pinene with the nitric acid exposed hematite and kaolinite surface (Figures S2 and S3). New spectral features produced from the reaction of α-pinene with nitrated hematite and kaolinite primarily are observed in the spectral regions extending from 2800 to 3050 cm^–1^ and 1450 to 1475 cm^–1^.^30,49,53,58^ Furthermore, strong absorptions at ∼3500 cm^–1^ (3507, 3472, 3355 cm^–1^) corresponding to the presence of different oxygenated organic compounds and hydrogen bonding were observed on the hematite surface. Therefore, the reactions of α-pinene with nitrated hematite and kaolinite surfaces suggest the formation of new surface-bound products through heterogeneous reactions in the dark and under dry conditions.

The spectral features of surfaces after the reaction of NO_2_(g) and α-pinene were different from those of from nitrated surfaces with α-pinene. In these experiments, α-pinene and NO_2_(g) were introduced to the IR cell with the mineral surface present. Then the reaction was allowed to occur over period of 4 h. After this time period, the infrared cell was evacuated. Unlike that seen for α-pinene alone, there remained adsorbed products on the surface following the evacuation. In particular, the spectrum of the hematite surface showed peaks at 2957, 2928, and 2876 cm^–1^ as well as at 1472 and 1440 cm^–1^, suggesting the presence of α-pinene and/or its derivatives on the surface. Additionally, the appearance of peaks at 1687 and 1215 cm^–1^ implies the formation of oxygenated pinene derivatives on the surface. For the kaolinite surface, the presence of oxygenated organic compounds was shown by the spectral features around 3745, 3779, ∼3500, and 1660 cm^–1^. The FTIR spectra of surface-bound products are provided in the Supporting Information (Figures S2 and S3). Furthermore, the products formed on these surfaces were analyzed with HRMS. In particular, the analysis of solvent extracted surface products provided was used to identify specific products.

**Figure 4:**
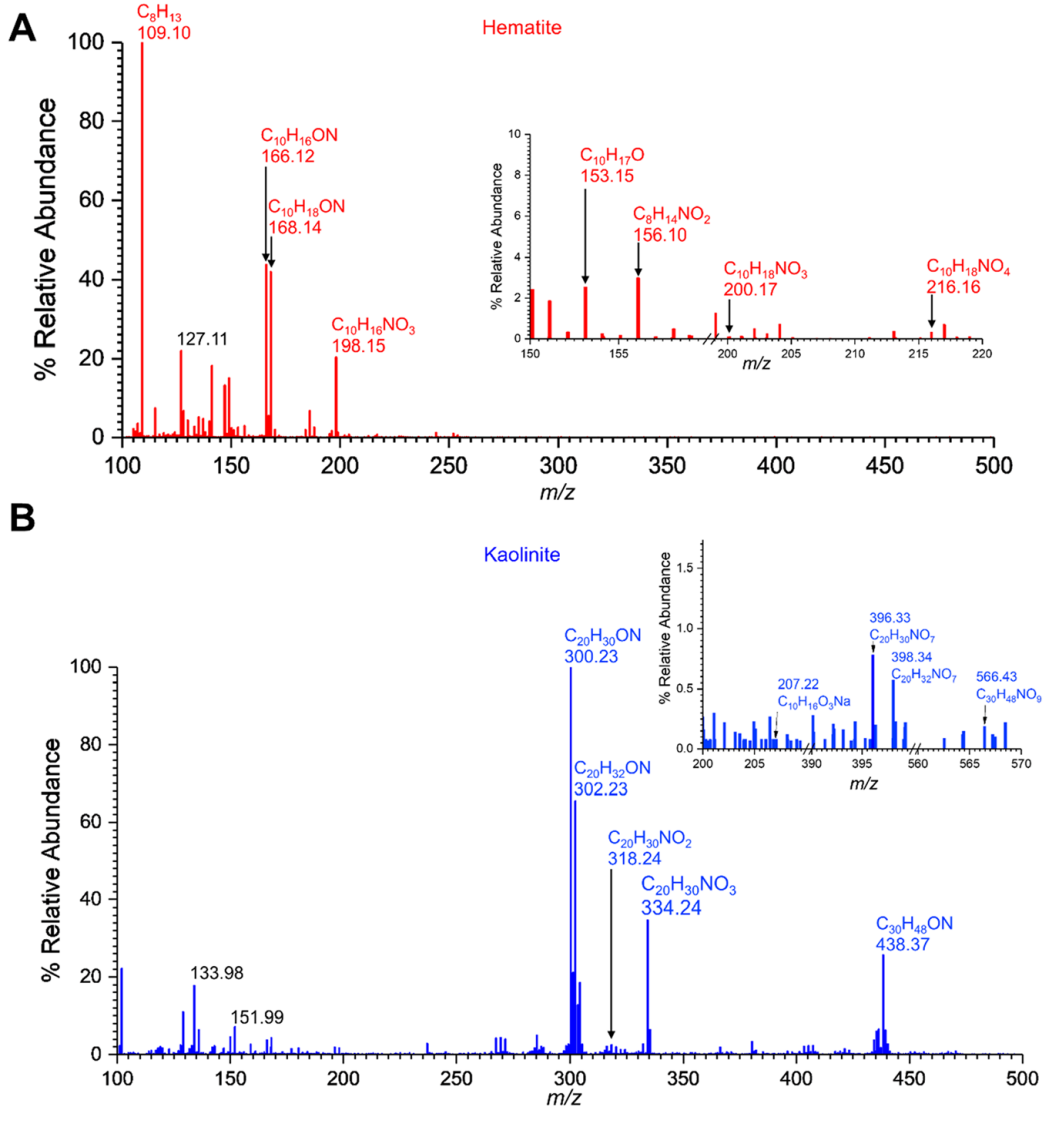
HRMS patterns in positive ESI mode, [M + H]^+^, of surface-bound products from the reactions of α-pinene with adsorbed nitrate (A) on hematite (B) on kaolinite.

To identify these surface-bound products formed and to understand possible reaction pathways, these products formed on mineral surfaces were extracted and analyzed via high-resolution mass spectrometry (HRMS). The proposed structures were further confirmed using tandem mass spectrometry (MS/MS). From the reaction of α-pinene with adsorbed nitrates on hematite, HRMS analysis suggested the formation of a series of ON compounds (Figure 4A, Table 2). Compound **3** (C_10_H_16_NO_3_, *m*/*z* = 198.15) and compound **4** (C_10_H_18_NO_3_, *m*/*z* = 200.17) were identified as the major ON compounds formed. The MS/MS analysis of compounds **3** and **4** confirmed the formation of fragments C_10_H_16_ON (*m*/*z* = 166.12) and C_10_H_18_ON (*m*/*z* = 168.14), respectively, among others. Compound **5** (C_10_H_18_NO_4_, *m*/*z* = 216.23) was identified with low relative intensity and with fragments *m*/*z* = 184.13 and 109.10 corresponding to C_10_H_18_O_2_N and C_8_H_13_ respectively. A smaller peak observed for compound **6** (C_10_H_17_O, *m*/*z* = 153.15) was assigned to α-pinene oxide. A possibility for compound **6** is isopinocamphone. However, the absence of a carbonyl peak ∼1740 cm^–1^ in the FTIR spectrum (Figure S2) eliminated this possibility. Furthermore, the formation of the fragment C_8_H_13_ (*m*/*z* = 109.10) from α-pinene was confirmed through MS/MS analysis. Additionally, a smaller peak at C_10_H_17_ (*m*/*z* = 137.13) was identified as a fragment of α-pinene oxide, which also can be assigned to unreacted α-pinene that may remain on the surface.

**Scheme 2:**
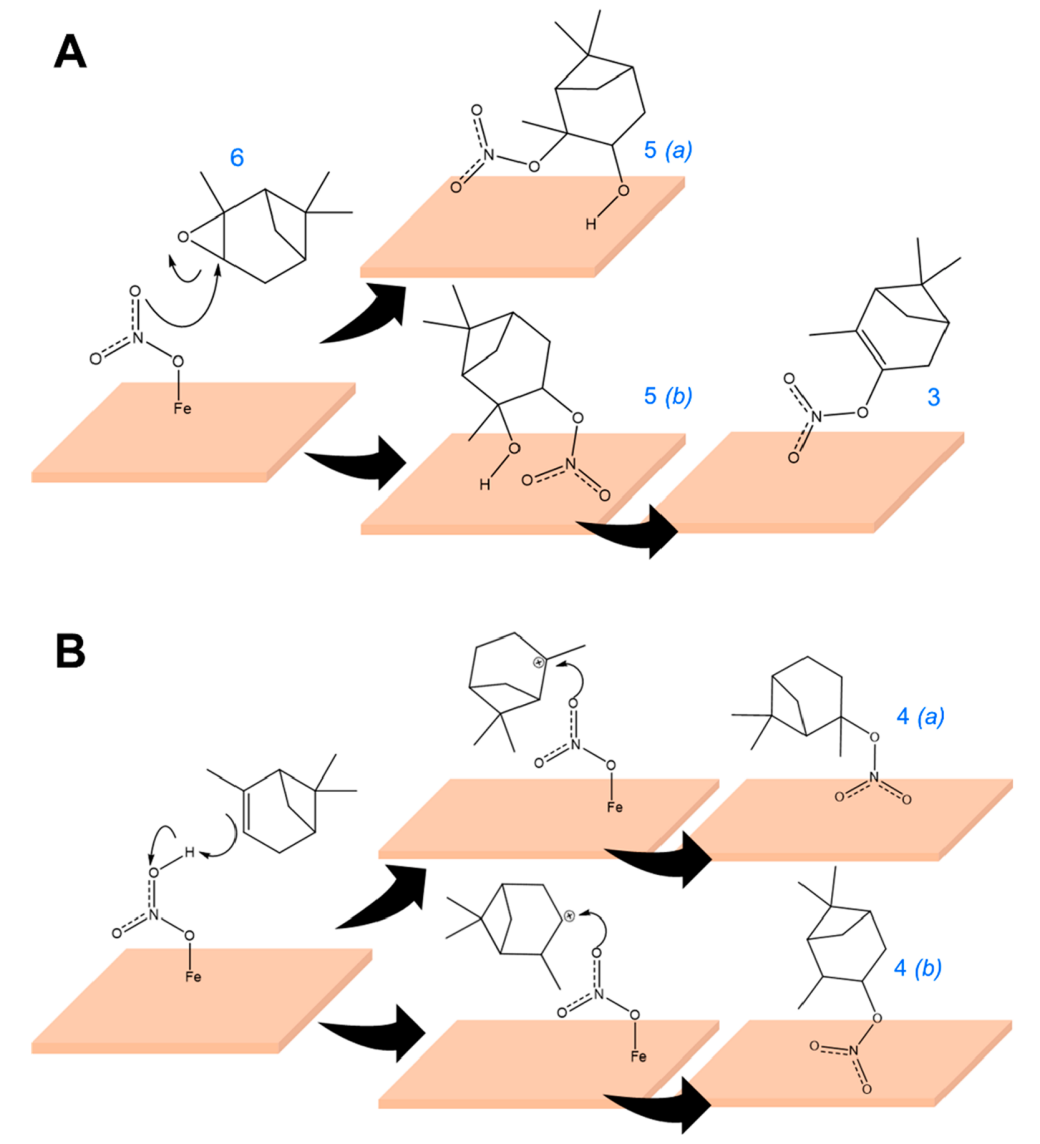
Proposed Mechanisms for the Formation of (A) Compounds **3** and **5** from Adsorbed Nitrate and α-Pinene Oxide, Formed from the Interaction of α-Pinene with Adsorbed Nitrates on Hematite and (B) Compound **4** from Adsorbed Nitrate and α-Pinene The structures **4**(*a*) and **4**(*b*) are structural isomers of compound **4**. Similarly, the structures **5**(*a*) and **5**(*b*) are structural isomers of compound **5**.

Scheme 2 illustrates the proposed formation pathways for the identified ON compounds. The formation of α-pinene oxide suggests that adsorbed nitrate on hematite oxidizes α-pinene. C_10_H_17_O (*m*/*z* = 153.15) was not observed in experiments with α-pinene adsorption on hematite, suggesting the epoxidation of α-pinene likely involves adsorbed nitrate, and perhaps iron-nitrate-redox chemistry.^59^ This is then followed by a nucleophilic attack from adsorbed nitrate on either carbon attached to the O atom on α-pinene oxide (electrophile) can form the two isomers of compound **5** (Scheme 2A). Compound **3** may form by dehydration of compound **5***(b)*. The formation of Compound **4** may occur via the interaction of π bonds in α-pinene with the oxygen atoms in adsorbed nitrates leading to the formation of a carbocation intermediate. The reaction of α-pinene π bonds with acid groups leading to carbocation formation was previously proposed.^51,60−62^ The formed carbocation then can react with adsorbed nitrate species on the hematite surface to form the isomers of compounds **4***(a)* or **4***(b)*, respectively (Scheme 2B**)**. Though the formation of both isomers is possible, a higher yield of the compound **4***(b)* is expected because of the higher stability of tertiary carbocations.

In contrast to lower molar mass ON compounds observed in the reactions of α-pinene with nitrated hematite, high molecular weight ON compounds were observed with nitrated kaolinite surfaces (Figure 4B). Among these were compound **7** (C_20_H_31_NO_7_, *m*/*z* = 398.34) and compound **8** (C_30_H_48_NO_9_, *m*/*z* = 566.43). These compounds were further confirmed by analyzing their MS/MS fragmentation patterns. For instance, the fragment C_30_H_48_ON (*m*/*z* = 438.37) was produced by the parent peak corresponding to compound **8** at 566.43. Apart from these ON compounds, the oxidation products, compound **2** (pinonaldehyde) and compound **9** (C_10_H_16_O_3_, *m*/*z* = 207.22, pinnonic acid), were observed in minor quantities.

Pinonaldehyde and its dimer were produced from α-pinene upon exposure to kaolinite surfaces. Therefore, a similar product formation can be expected from α-pinene reactions on nitrated kaolinite surfaces. The detection of compound **9** (pinonic acid) suggests at least some of the produced pinonaldehyde further oxidizes on the nitrated kaolinite surface. Therefore, it can be speculated that the aldehyde end of the produced pinonaldehyde, pinonaldehyde dimer, and pinonaldehyde trimer oxidizes to form a carboxylic acid end in the presence of nitrated kaolinite surfaces. The adsorbed nitrate groups react with this carboxylic acid end to form PAN-analogues (peroxy acyl nitrate) of these compounds, resulting the observed compounds **7** and **8** in this study. Furthermore, this observation suggests more α-pinene was oxidized to form pinonaldehyde in the presence of adsorbed nitrate on kaolinite, thereby facilitating its trimerization. Thus, again, these results underline the important role of the surface chemical reactions in formation of ON from α-pinene in the dark and low RH environments.

**Figure 5:**
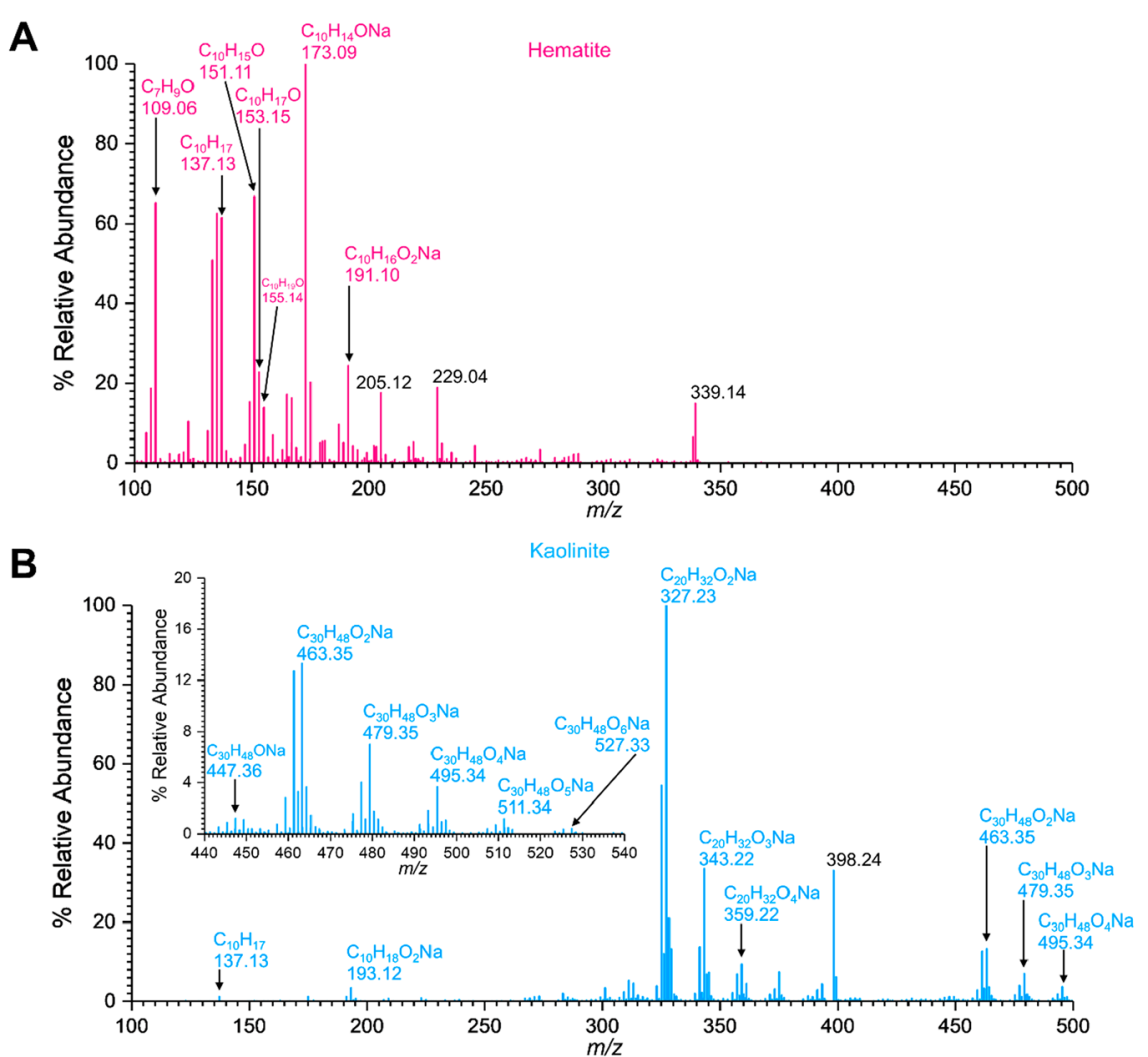
HRMS patterns in positive ESI mode, [M + H]^+^, of surface-bound products from the reactions of α-pinene with NO_2_ (A) on hematite (B) on kaolinite.

In gas-phase reactions of α-pinene with NO_2_(g) in the presence of hematite, the formation of a mixture of oxidation products of α-pinene was observed (Figure 5A, Table 2). Here, compounds **2** (pinonaldehyde) and **10** (C_10_H_19_O, *m*/*z* = 155.14, isopinocampheol) and *m*/*z* = 153.15 corresponding to C_10_H_17_O were identified. However, no evidence of ON formation was seen. Therefore, the major identified SOA products from the reaction of gas-phase NO_2_ with α-pinene in the presence of hematite are oxidation products of α-pinene. In contrast to reactions of adsorbed nitrate on hematite with α-pinene, here the peak at *m*/*z* = 153.15 corresponding to C_10_H_17_O is most likely several species of similar mass including α-pinene oxide, isopinocamphone, and α-pinene derived alcohols such as verbenol.^63−65^ This is because of the complex mass fragmentation pattern produced with a strong fragment at C_7_H_9_O (*m*/*z* = 109.06), along with C_10_H_17_ (*m*/*z* 137.13) with many smaller peaks for fragments below *m*/*z* = 100, all corresponding to possible isomers of C_10_H_17_O.

In contrast to the variety of surface-bound oxidation products seen from the hematite surface, for kaolinite only compound **1** (pinonaldehyde dimer), compound **2** (pinonaldehyde), and compound **11** (C_30_H_48_O_6_, *m*/*z* = 527.33, pinonaldehyde trimer) were observed. Therefore, it is likely that the presence of NO_2_ in the gas phase causes further oxidation of α-pinene to pinonaldehyde, which then leads to trimerization. Similar evidence of oligomerization of pinonaldehyde has been observed previously.^66^

The oxidation products of α-pinene are widely seen in the atmosphere and have shown to readily form in the presence of typical atmospheric oxidants.^4,6,25,26^ However, their formation in the presence of gas-phase NO_2_ and in the absence of other oxidizers such as O_3_ and HO^•^ was not previously observed. Therefore, it is proposed that the α-pinene oxidation by NO_2_ can occur in the presence of mineral surfaces, thereby producing the observed oxidation products of α-pinene. The formed oxidation products adsorb onto the mineral surface, thus increasing SOA formation. Additionally, these findings underscore the importance of particle mineralogy and the role that different mineral dust surfaces play in this chemistry.

## 4 Conclusions and Atmospheric Implications

The reactions of α-pinene on hematite and kaolinite surfaces at 296 K yield various ON and oxidation products of α-pinene. In particular, α-pinene oxidized to pinonaldehyde followed by dimerization on the kaolinite surface, whereas no such product formed on hematite. When NO_2_ is present or the surface is first exposed to HNO_3_, multiple ON products, as well as oxidation products, were identified with both mineral surfaces. The proposed formation pathways of these ON compounds occur via an adsorbed nitrate species. In the case of kaolinite, the adsorbed nitrates react with pinonaldehyde and its oligomers to produce a carboxylic acid end which then reacts with adsorbed nitrates to form PAN-type compounds. In contrast to kaolinite, adsorbed nitrate reacts with α-pinene on hematite to yield lower molar mass ON compounds. Furthermore, the fate of gas-phase reaction products of the gas-phase α-pinene plus NO_2_ reaction was also investigated. These gas-phase reactions lead to several oxidized α-pinene derivatives that form strong interactions with these mineral surfaces. Eleven unique surface-bound products were identified.

Overall, this study shows how mineral dust aerosol not only has the potential to act as seed particles for SOA formation by facilitating adsorption of lower volatility oxidized organic compounds but also can provide a reactive surface for reactions to occur that is mineral specific, leading to the formation of ON and other SOA components. Additionally, these findings suggest further investigation of the heterogeneous and multiphase mineral oxide-mediated chemistry of these abundant monoterpenes under various environmental conditions, such as high RH environments, daytime with abundant sunlight, and other mineral surfaces (e.g., other metal oxides, other clay types). These types of studies will provide the basis for further understanding of the role of mineral dust aerosol on air quality, climate change, and human health.

## Supporting Information Available

Figure S1, FTIR spectra of adsorbed nitrates on hematite and kaolinite surfaces following reaction with nitric acid; Figure S2, FTIR spectra of strongly bound surface products on hematite following reactions with nitrogen oxides; Figure S3, FTIR spectra of strongly bound surface products on kaolinite following reactions with nitrogen oxides (PDF)
