# Supplementary material for: Heterogeneous Reactions of α-Pinene on Mineral Surfaces: Formation of Organonitrates and α-Pinene Oxidation Products
Source: J Phys Chem A. 2022 Jun 16;126(25):4068–79. doi: 10.1021/acs.jpca.2c02663 (PMC9251774; doi:10.1021/acs.jpca.2c02663)
Supplement: Supplementary file 1 — jp2c02663_si_001.pdf [file jp2c02663_si_001.pdf]

Supporting Information

for

Heterogeneous Reactions of α-Pinene on Mineral Surfaces: Formation of Organonitrates and α-Pinene Oxidation Products

Eshani Hettiarachchi and Vicki H. Grassian*

Department of Chemistry and Biochemistry, University of California San Diego, 9500 Gilman Drive, La Jolla, California, 92093 United States.

*Author to whom correspondence should be addressed (vhgrassian@ucsd.edu)

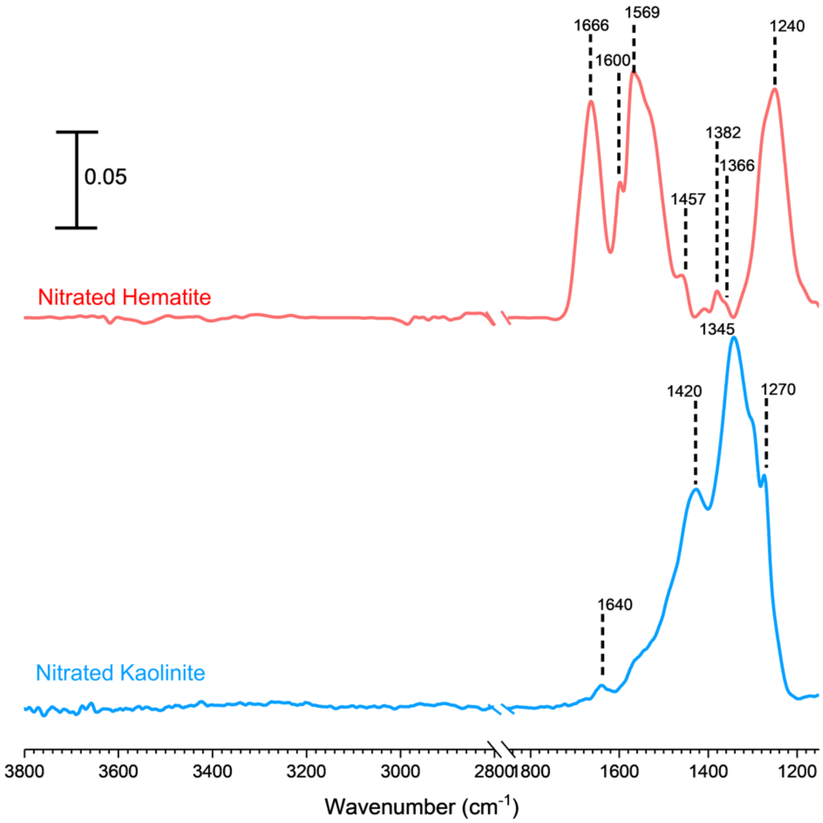

Figure S1: FTIR spectra of nitrated mineral surfaces. Several coordination modes of adsorbed nitrate are observed following exposure to HNO_3. Among these, monodentate, bidentate, bridging nitrate, and water solvated nitrate were observed. These observed peaks are in agreement with the previous studies conducted for HNO_3 and NO_2 on mineral surfaces.[1–6] It can be seen that nitrate coordination differs on these two surfaces.[2,7]

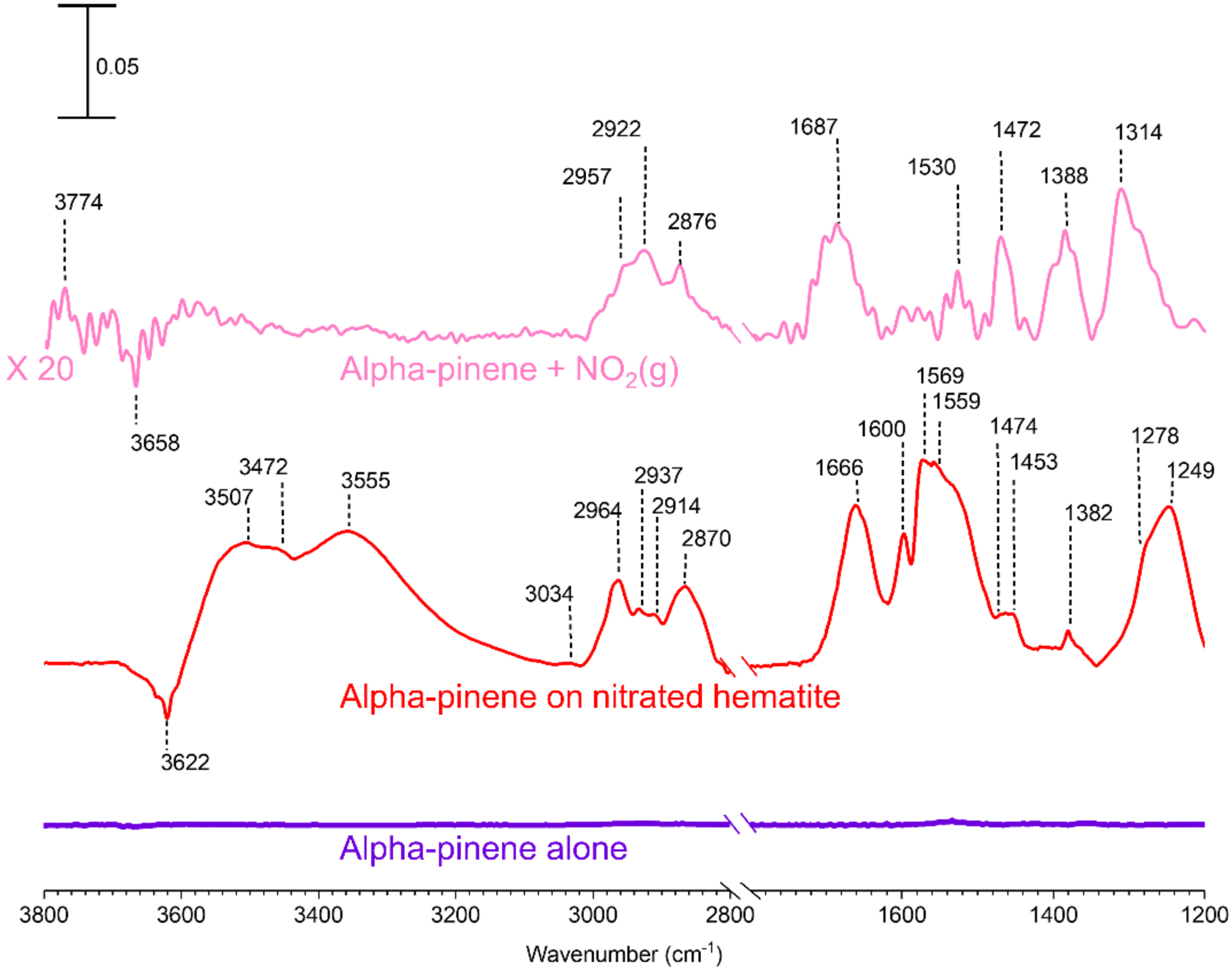

Figure S2: FTIR spectra of strongly bound products from the reaction of α-pinene with adsorbed nitrates, and the reaction of α-pinene with NO_2(g) on hematite following evacuation. Several new peaks in the regions of 2800 – 3050 cm^{-1} and 1450 – 1475 cm^{-1} were observed, suggesting the presence of C-H bonds on the surface.[3,8–10] Furthermore, strong broad bands at ~3500 cm^{-1} (3507 cm^{-1}, 3472 cm^{-1}, 3355 cm^{-1}) corresponding to different oxygenated organic compounds and hydrogen bond networks were observed. The spectral features of surfaces after gas-phase reaction of NO_2(g) and α-pinene were different from those of nitrated surfaces with α-pinene. Despite the relatively lower intensity, the spectrum for hematite surface showed peaks at 2957 cm^{-1}, 2928 cm^{-1}, 2876 cm^{-1} as well as at 1472 cm^{-1} and 1440 cm^{-1} suggesting the presence of α-pinene and/ or its derivatives on the surface. Additionally, the appearance of peaks at 1687 cm^{-1} and 1215 cm^{-1} implies the formation of oxygenated pinene derivatives on the surface.

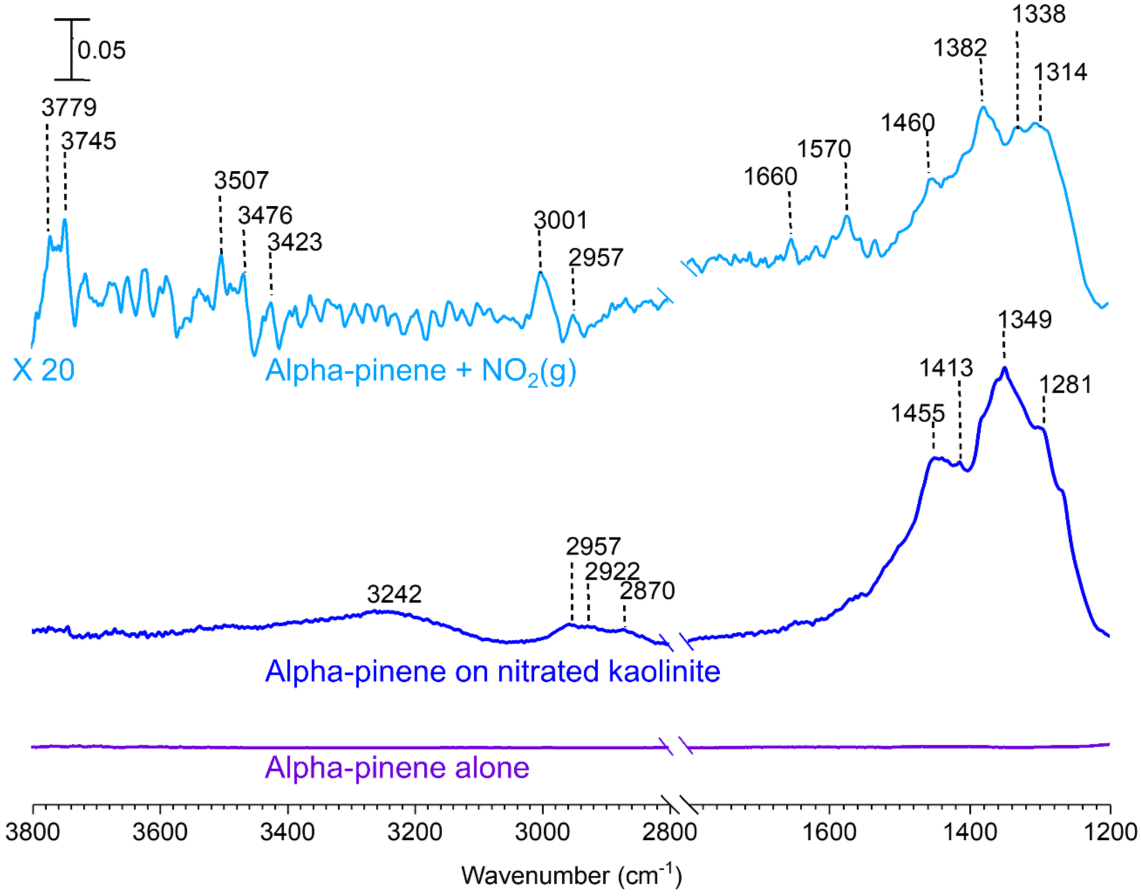

Figure S3: FTIR spectra of strongly bound products from the reaction of α-pinene with adsorbed nitrates, and from the reaction of α-pinene with NO_2(g) on kaolinite following evacuation. Several new peaks in the regions of 2800 – 3050 cm^{-1} were observed, suggesting the presence of C-H bonds on the surface.[3,8–10] The spectral features of surfaces after the reaction of NO_2(g) and α-pinene were different from those of from nitrated surfaces with α-pinene. For kaolinite surfaces, the presence of oxygenated organic compounds was shown by the spectral features around 3745 cm^{-1}, 3779 cm^{-1}, ~3500 cm^{-1}, and 1660 cm^{-1}.

References

(1) Underwood, G. M.; Miller, T. M.; Grassian, V. H. Transmission FTIR and Knudsen Cell Study of the Heterogeneous Reactivity of Gaseous Nitrogen Dioxide on Mineral Oxide Particles. *J. Phys. Chem. A* **1999**, *103* (31), 6184–6190.

(2) Goodman, A. L.; Bernard, E. T.; Grassian, V. H. Spectroscopic Study of Nitric Acid and Water Adsorption on Oxide Particles: Enhanced Nitric Acid Uptake Kinetics in the Presence of Adsorbed Water. *J. Phys. Chem. A* **2001**, *105*, 6443–6457.

(3) Liu, C.; Ma, Q.; Liu, Y.; Ma, J.; He, H. Synergistic Reaction between SO_2 and NO_2 on Mineraloxides: A Potential Formation Pathway of Sulfate Aerosol. *Phys. Chem. Chem. Phys.* **2012**, *14* (5), 1668–1676.

(4) Miller, T. M.; Grassian, V. H. Heterogeneous Chemistry of NO_2 on Mineral Oxide Particles: Spectroscopic Evidence for Oxide-Coordinated and Water-Solvated Surface Nitrate. *Geophys. Res. Lett.* **1998**, *25* (20), 3835–3838.

(5) Hixson, B. C.; Jordan, J. W.; Wagner, E. L.; Bevsek, H. M. Reaction Products and Kinetics of the Reaction of NO_2 with γ-Fe_2O_3. *J. Phys. Chem. A* **2011**, *115* (46), 13364–13369.

(6) Angelini, M. M.; Garrard, R. J.; Rosen, S. J.; Hinrichs, R. Z. Heterogeneous Reactions of Gaseous HNO_3 and NO_2 on the Clay Minerals Kaolinite and Pyrophyllite. *J. Phys. Chem. A* **2007**, *111* (17), 3326–3335.

(7) Baltrusaitis, J.; Schuttlefield, J.; Jensen, J. H.; Grassian, V. H. FTIR Spectroscopy Combined with Quantum Chemical Calculations to Investigate Adsorbed Nitrate on Aluminium Oxide Surfaces in the Presence and Absence of Co-Adsorbed Water. *Phys. Chem. Chem. Phys.* **2007**, *9* (36), 4970.

(8) Huang, L.; Frank, E. S.; Riahi, S.; Tobias, D. J.; Grassian, V. H. Adsorption of Constitutional Isomers of Cyclic Monoterpenes on Hydroxylated Silica Surfaces. *J. Chem. Phys.* **2021**, *154* (12), 124703.

(9) Chase, H. M.; Ho, J.; Upshur, M. A.; Thomson, R. J.; Batista, V. S.; Geiger, F. M. Unanticipated Stickiness of α-Pinene. *J. Phys. Chem. A* **2017**, *121* (17), 3239–3246.

(10) Ho, J.; Psciuk, B. T.; Chase, H. M.; Rudshteyn, B.; Upshur, M. A.; Fu, L.; Thomson, R. J.; Wang, H.-F.; Geiger, F. M.; Batista, V. S. Sum Frequency Generation Spectroscopy and Molecular Dynamics Simulations Reveal a Rotationally Fluid Adsorption State of α-Pinene on Silica. *J. Phys. Chem. C* **2016**, *120* (23), 12578–12589.
